# Variants in CAPN3 Causing Autosomal Dominant Limb–Girdle Muscular Dystrophy Combined With Calpain-3 Deficiency

**DOI:** 10.1155/humu/9301465

**Published:** 2025-03-29

**Authors:** Thomas Krag, Emily Nasho, Lauren Brady, Camille Verebi, France Leturcq, Edoardo Malfatti, Morten Duno, Mark Tarnopolsky, John Vissing

**Affiliations:** ^1^Copenhagen Neuromuscular Center, Copenhagen University Hospital Rigshospitalet, University of Copenhagen, Copenhagen, Denmark; ^2^Neuromuscular and Neurometabolic Clinic, McMaster University Medical Centre, Hamilton, Canada; ^3^Service de Medecine Genomique, Maladies de Systeme et d'Organe-Federation de Genetique et de Medecine Genomique, DMU BioPhyGen, APHP Centre-Universite Paris Cite-Hopital Cochin, Paris, France; ^4^Universite Paris Est, U955, IMRB, INSERM, APHP, Centre de Reference de Pathologie Neuromusculaire Nord-Est-Ile-de-France, Filnemus, Henri Mondor Hospital, Paris, France; ^5^Department of Clinical Genetics, Copenhagen University Hospital Rigshospitalet, Copenhagen, Denmark

**Keywords:** autosomal dominant calpainopathy, calpain-3, limb–girdle muscular dystrophy Type D4

## Abstract

**Abstract:** Limb–girdle muscular dystrophy Type 2A/R1 or calpain-3 deficiency is the most common autosomal recessive limb–girdle muscular dystrophy. However, in recent years, autosomal dominant cases and families with calpain-3 deficiency have been reported, and there is an emerging interest in looking for single variants in the calpain-3 gene in mildly to moderately affected patients with limb–girdle muscular dystrophy without biallelic gene variants in *CAPN3*. Here, we report four cases with creatine kinase levels above 1500 U/L, mild-to-moderate proximal weakness, waddling gait, and scapular winging. Two patients, a son and his father, are heterozygous for the *CAPN3* variant c.304C>T; p.(Pro102Ser), which has previously been reported in patients with compound heterozygous variants in *CAPN3*. The third and fourth patients were heterozygous for c.1371C>G; p.(Asn457Lys) and c.1490C>T; p.Ala497_Glu508del, respectively, neither of which has been reported before. All four patients had a near-complete loss of calpain-3 as determined by western blotting. While inherited autosomal dominant calpainopathy has now been firmly established, additional single cases of dominant calpainopathy are likely to emerge; some will be associated with clinical findings from parents or siblings, while others will arise from spontaneous mutations, but nevertheless with similar clinical findings of mild-to-moderate proximal weakness, increased level of creatine kinase, and near-complete loss of calpain-3 protein in affected individuals. This report expands the known number of variants causing dominant calpainopathy from 8 to 11 that appears to exclusively reside in two out of four domains that make up calpain-3. This information could aid in determining whether a *CAPN3* variant of unknown significance is pathological.

## 1. Introduction

**Figure 1:**
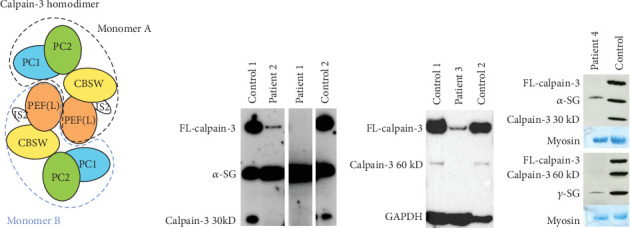
Calpain-3, patient calpain-3 levels, and calpain-3 AD variant distribution. (a) The dimerized calpain-3 consists of four functional domains each. The proteolytic Cores 1 and 2 (PC1/PC2), calpain-type beta-sandwich (CBSW), and the PEF(L) dimerization domain. (b) Western blot of calpain-3 (FL full length) shows that this is barely detectable in Patients 1 and 2 compared to controls; *α*-sarcoglycan (*α*-SG) is used as a loading control. (c) Western blot of calpain-3 from Patient 3 also shows barely detectable calpain-3 compared to controls; glyceraldehyde-3-phosphate dehydrogenase (GAPDH) was used as a loading control. (d) Western blot of calpain-3 from Patient 4 also shows barely detectable calpain-3 compared to controls; myosin heavy chain (myosin) was used as a loading control. Western blotting was carried out at two sites (Paris and Copenhagen) in three separate runs as shown over a period of 3 years.

Limb–girdle muscular dystrophy Type 2A/R1 is the most common autosomal recessive limb–girdle muscular dystrophy with a prevalence of around 1:100,000 with minor regional differences [1]. It is caused by biallelic pathogenic variants in the *CAPN3* gene coding for the calpain-3 protein, a protease bound to the sarcomeric protein titin and involved in sarcomere turnover as part of maintenance and muscle regeneration [2–4]. Calpainopathy causes progressive proximal weakness, and most patients have elevated creatine kinase (CK) levels [5]. An autosomal dominant calpainopathy was first described in 2016 [6], and since then, eight heterozygous variants in *CAPN3* have been associated with autosomal dominant limb–girdle muscular dystrophy Type D4 (LGMD D4) [7–13]. A common feature of patients with autosomal dominant variants is a later onset and milder form of limb–girdle muscular dystrophy with slower progression compared to the recessive form. Proper determination of LGMD D4 requires western blotting for the calpain-3 protein level in a muscle biopsy as likely only a subset of known (and yet unknown) *CAPN3* variants can give rise to the dominant form. Hence, it may be helpful to determine if a variant in *CAPN3* is more or less likely to cause LGMD D4. Unfortunately, this cannot easily be inferred from patients with LGMD2A/R1 homozygous for *CAPN3* pathogenic variants because even these patients may have semi- to normal levels of calpain-3. The inactive and active forms of calpain-3 are homodimers consisting of four domains, the protease Cores 1 and 2 (PC1/PC2), the calpain-type beta-sandwich (CBSW) that is important for autolytic activity, and the penta-EF hand that is responsible for dimerization (Figure 1). As inactive, the homodimer is bound to the giant sarcomeric protein, titin. For activation to occur, the calpain-3 dimer needs to bind calcium ions, leading to an internal autolytic cleavage, rearrangement of calpain-3 domains through an intermolecular complementation (iMOC), and eventually the formation of the proteolytically active enzyme [14, 15]. As treatments are being developed, more information about genotype–phenotype relationships is needed to avoid that treatment-related calpain-3 expression being proteolyzed before it can attain its proper function. Here, we report three new *CAPN3* variants, associated with LGMD D4, found in a father and son, and two unrelated, single patients along with the clinical findings.

## 2. Case Report

Informed consent for diagnostic workup on muscle and blood samples was obtained for all individuals, and all investigations were carried out in accordance with the Declaration of Helsinki.

Patient 1 is a 62-year-old male of Northern European descent who presented at 49 years of age with an incidental finding of elevated CK (1500–5000 U/L, normal < 165 U/L) on routine bloodwork. He reported minor myalgias at night but no history of weakness or gait abnormalities. Physical exam revealed bilateral calf hypertrophy with otherwise normal muscle strength (MRC scale) for proximal and distal muscles of the upper and lower extremities. EMG studies revealed an early recruitment pattern and small brief motor unit action potentials in the deltoid muscle. A muscle biopsy of the *vastus lateralis* did not show any pathological abnormalities or abnormal staining on light microscopy. Electron microscopy showed a few ring fibers and a slight tendency of the mitochondria to accumulate in the subsarcolemmal location. By 55 years of age, he had developed mild proximal weakness and a waddling gait. The patient reports that starting in his mid-30s, he had noted a waddling gait after prolonged walking, and in the past 7 years, he needed to use the railing for stairs. He can lift his arms overhead but cannot perform sustained activities (e.g., painting overhead). Neurological examination at 59 years of age showed mild scapular winging with weakness of forward arm flexion, hip adduction, and hip abduction (MRC = 4/5). All other proximal and distal muscles had normal strength (MRC = 5/5). Spirometry was normal (FVC = 85% predicted). Upon further reflection, the patient believed that his father, paternal grandfather, paternal aunt, and two brothers reportedly exhibited symptoms of proximal muscle weakness (i.e., late-onset waddling gait); however, they were not evaluated.

Patient 2 is the son of Patient 1. He was first evaluated at 27 years of age after he was found to have an elevated CK (1700 U/L, normal < 165 U/L). He reported some shoulder fatigue in his late teens but had a normal neurological examination, normal muscle strength for major muscle groups proximally and distally in the upper and lower extremities, and normal spirometry (FVC = 89% predicted). EMG of the medial gastrocnemius and vastus medialis showed no clear spontaneous activity with only a minimal increase in insertional activity and rare positive sharp waves. A muscle biopsy of the *vastus lateralis* showed denervation without reinnervation and minimal microangiopathy. Electron microscopy showed a slight increase in the number of lipid droplets. Examination at 29 years of age found mild hip flexor weakness (4+/5) and mild scapular weakness (4+/5) on the MRC scale. He reported experiencing overhead fatiguability and myalgias. A comprehensive neuromuscular panel identified a variant in *CAPN3* (c.304C>T; p.(Pro102Ser), NM_000070.2, rs2141102957) in both father and son. No second variant was identified. This variant has been observed in an individual with a limb–girdle muscular dystrophy phenotype and suspected recessive inheritance, but no detailed information has been given [16]. PolyPhen-2 and MutationTaster predict the variant to be probably damaging (score 0.999/1) and disease-causing due to loss of catalytic function. Both CADD and REVEL predict the variant to be deleterious. Western blotting for calpain-3 expression demonstrated that the level of calpain-3 was very low (estimated < 5%) in both patients compared to healthy control subjects (Figure 1b).

Patient 3 is a 54-year-old female of Northern European descent with a history of left calf atrophy and muscle fatigue and some shortness of breath on exertion. Her CK was found to be elevated at ~1000 U/L (normal < 165 U/L). A neurological exam did not identify any scapular winging or waddling gait. There was no clear history of myalgias or lower back pain, but she had developed brachial neuritis in her right arm several years prior to the examination. EMG showed a mild increase in insertional activity in both the left and right medial gastrocnemius muscles. MRC testing was 5/5 muscle strength for all muscles. Her father was reported to have had similar symptoms and a waddling gait and had been evaluated in the same clinic almost 20 years prior. His CK was also elevated, and he was diagnosed with an unknown type of limb–girdle muscular dystrophy but since deceased. Initial targeted analysis by a focused gene panel for neuromuscular disorder identified a novel heterozygous variant in *CAPN3*, c.1371C>G; p.(Asn457Lys). Subsequent WGS and comprehensive analysis did not reveal other aberrations in *CAPN3* nor any alternative genetic explanation. The variant was found in gnomAD (1/833088 alleles) but not in LOVD or ClinVar. It has been submitted by the authors to LOVD (https://databases.lovd.nl/shared/individuals/00448363). PolyPhen-2 and MutationTaster predict the variant to be probably damaging (score 1.000/1) and disease-causing. Both CADD and REVEL predict the variant to be deleterious. Western blotting for calpain-3 demonstrated that the level was very low (estimated < 5%) compared to healthy control subjects (Figure 1c).

Patient 4 is a male, born to nonconsanguineous allegedly healthy parents from Réunion island. He denies the presence of muscle symptoms in his parents, siblings, and children. The family members were not available for clinical assessment or genetic studies. At the age of 57, he presented with myalgias in his thighs and difficulties in standing. CK level was 2000 U/L and a first muscle biopsy was performed suggesting polymyositis. The patient was treated with immunosuppressive treatment (azathioprine, methotrexate) without any effect. The cardiac and respiratory workup did not reveal anything abnormal. At the age of 60, he presented with a waddling gait and a CK level of 5000 U/L. At the age of 62, muscle MRI demonstrated fibrofatty substitution of paraspinal, gluteal, and hamstring muscles, and at the age of 64, a new muscle biopsy of *vastus lateralis* demonstrated internalized nuclei, few necrotic regenerating fibers, and hypercontracted fibers without inflammation. At the last clinical evaluation at the age of 66, the patient presented with asymmetric scapular winging, waddling gait, and MRC testing showing weakness in the psoas (4/5), gluteal (4/3), and hamstring (3/3) muscles. Western blotting for calpain-3 demonstrated a near-complete loss of protein compared to a healthy control subject (Figure 1). Initial gene panel analysis revealed the heterozygous presence of a novel variant c.1490C>T; p.(Ala497Val) (rs749969359) in *CAPN3*. Subsequent WGS and comprehensive analysis confirmed the sole heterozygous presence of the variant and did not reveal any other potential genetic explanation. Splicing predictors such as SPiP and SpliceAI visual predicted the creation of an alternative splice site donor [17, 18]. cDNA analysis from muscle RNA confirmed the prediction and revealed an abnormal transcript deleted for the 36 terminal nucleotides of Exon 11 (r.1490_1525del) alongside the full-length transcript. The deletion creates an *in-frame* transcript lacking 12 codons (p.(Ala497_Glu508del)). The variant was not found in gnomAD, LOVD, and ClinVar and has been submitted by the authors to LOVD (https://databases.lovd.nl/shared/variants/000060182#00004330). The cDNA analyses furthermore show an approximately 1:1 between the two alleles arguing against the activation of nonsense-mediated mRNA decay and thus the translation of the abnormal transcript. The patient had decided not to disclose his calpain-3-related disease to extended family, so no further segregation analysis could be performed.

## 3. Discussion

In this case series, we demonstrate three novel heterozygous *CAPN3* variants associated with autosomal dominant LGMD D4. All variants result in a nearly complete loss of both mutated and wild-type calpain-3 on western blot. This is consistent with previous reports of patients with autosomal dominant calpainopathy. In the case of Patients 1 and 2, there is vertical transmission of dominant calpainopathy. The variant has initially been described in a patient with a presumed biallelic *CAPN3* defect, but no detailed information is provided or possible parental phenotype [16]. Dominant *CAPN3* defects are generally clinically much milder than the recessive counterpart and thus can be overlooked. The first variant undisputably associated with a dominant phenotype was likewise initially described in LGMD2A/1R. In Patient 3, the disease is likely inherited in an autosomal dominant manner since the father and paternal relatives exhibited the same symptoms. For Patient 4, it remains unclear whether the variant is inherited or de novo. cDNA analysis confirms in creation of an alternative 5⁣′ splice site predicting a protein deleted for 12 amino acids. The initial variant associated with dominant LGMD D4, likewise, created a protein deleted for seven amino acids pointing to misfolding of calpain-3 as a possible mechanism. The variant in Patients 1 and 2 is likely to affect Ca^2+^ binding in PC1, which is a prerequisite for catalytic activity and therefore is likely to lead to loss of catalytic activity. In Patients 3 and 4, the variants are in the CBSW domain at or near the calmodulin Binding Sites 1 and 2, likely affecting the autolytic activity. For some of the previously known variants leading to dominant calpainopathy, the loss of catalytic/autolytic activity has either been determined experimentally or been inferred from structural analysis of the conformational changes due to the variants [9, 11, 19]. However, even if it is known which proteolytic part of calpain-3 is affected, catalytic or autolytic, there is yet no evidence to explain the dominant negative effect of any of the variants. The reason for this may lie in the complex activation of calpain-3, where PC2 is cleaved at IS1 and reassembled with PC1, and with a dual-mode iMOC that may work in *trans* on other calpain-3 dimers and bind both cleaved PC1/PC2 and full-length calpain-3 [15]. Clearly, this also suggests that the dominant negative effect can take place in more than one way. The *trans*-effect, rather than a *cis*-effect within the dimer, has been proposed to explain why patients with compound heterozygous variants often are milder affected than the corresponding homozygous variant of either type [3, 20]. Due to the iMOC, and within the boundaries of the PC1/PC2 fragment, a variant in this cleaved part can be swapped for a normal one from the other variant, basically creating a fully normal activated calpain-3. The dynamics of this process is likely determined by multiple factors but poorly understood in vivo. While several studies have investigated the genotype–phenotype relationship among patients homozygous for autosomal recessive variants, this knowledge may not be applicable to determine which variants may cause dominant calpainopathy and predict the resulting phenotype. However, as more dominant variants are identified and reported, we may be able to infer how the dominant effect leads to this near-total loss of calpain-3 protein. A retrospective clinical study that is supposed to finish in late 2025 “Clinical and Biochemical Features for the Identification of Dominant Calpainopathies (DOM-CAL)” (https://clinicaltrials.gov/study/NCT05956132) should expand the number of known variants associated with autosomal dominant calpainopathy and hopefully lead to more research to understand some of the poorly understood facets of calpain-3 function. Even if we do not know the exact mechanism of loss of calpain-3 in autosomal dominant patients, it should be absolutely clear that as the near-complete loss of calpain-3 affects both the variant and normal calpain-3, any treatment-mediated expression of calpain-3 in muscle may just be degraded as it is being expressed. Thus, apart from stability issues, a future AAV-based calpain-3 treatment may not benefit patients with autosomal dominant calpainopathy, and a separation of autosomal dominant and recessive patients with calpain-3 deficiency should be considered if preparing a clinical trial. Finally, it must be emphasized that this is a case study with limited genetic data but reporting an interesting observation on CAPN3 expression that will contribute to a better understanding of dominant calpainopathy.

As a conclusion, we have here reported four patients with symptoms of limb–girdle muscular dystrophy and heterozygous for variants in the *CAPN3* gene leading to LGMD D4. This study has expanded the number of known variants causing dominant calpainopathy from 8 to 11.

## Data Availability

Most of the data analyzed during this study can be found within the published article. Any additional raw data are available from the corresponding author on reasonable request.
